# The Complex (Organic) Puzzle of the Formation of Hydrogen Cyanide and Isocyanide on Interstellar Ice Analogues

**DOI:** 10.1021/acs.jpclett.4c01537

**Published:** 2024-07-25

**Authors:** Joan Enrique-Romero, Thanja Lamberts

**Affiliations:** † Leiden Institute of Chemistry, Gorlaeus Laboratories, Leiden University, PO Box 9502, 2300 RA Leiden, The Netherlands; ‡ Leiden Observatory, Leiden University, P.O. Box 9513, 2300 RA Leiden, The Netherlands

## Abstract

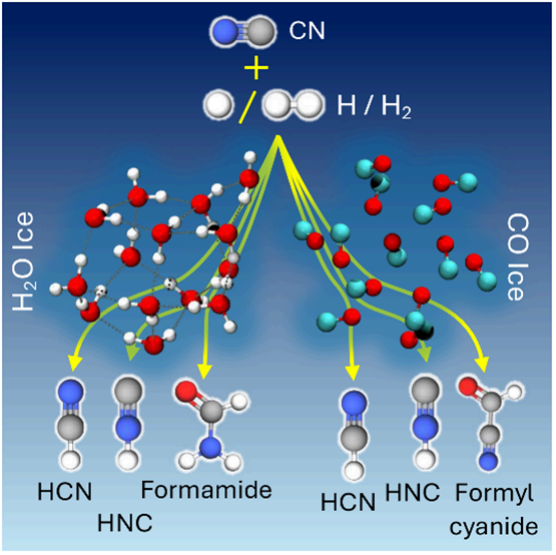

Aiming to constrain the surface formation of HCN and HNC in the dense interstellar medium on ice-covered dust grains, we investigate the interaction of CN radicals with H_2_O and CO ices and their subsequent reactivity with H and H_2_. CN radicals can physisorb on both ices. However, on H_2_O ice, a hemibond formation is the most common binding mode, while on CO ice, the CN–CO van der Waals complex can form NCCO with a small energy barrier. We show low barrier or barrierless pathways to the formation of HCN and HNC for the reaction H + CN on both ices. Reactivity with H_2_ involves activation energy barriers to form HCN, which may be overcome by quantum tunneling, while HNC formation is unlikely. The formation of HCN and HNC competes with the formation of NH_2_CHO on H_2_O and HCOCN on CO.

Nitriles are pivotal species in the development of prebiotic chemistry, and therefore are considered to play an important role in the emergence of life in the Universe.^1−3^ Their simplest representatives, HCN and HNC, are regarded as important parent species of interstellar complex organic molecules^4,5^ (iCOM, e.g., CH_3_NH_2_), cyanopolyynes^6^ (e.g., C_3_N) and anion–cation pairs^7^ on interstellar ices (e.g., the NH_4_^+^CN^–^ salt). Furthermore, historically, HCN played a key role experimentally in the formation of prebiotic molecules, e.g., the Strecker synthesis,^8^ the Miller-Urey experiments,^9,10^ and formation of amino acids.^11,12^

As a consequence of the large dipole moments, HCN and HNC are frequently detected in a wide range of environments in the interstellar medium including diffuse clouds,^13,14^ dense clouds,^15^ star forming regions,^16,17^ shocked regions,^18^ protoplanetary disks,^19^ comets^20,21^ and even external galaxies.^22^ Both molecules are routinely used to trace dense gas regions, precisely the places where stars and planets form,^16^ and their abundance ratio is a gas temperature tracer.^23^

Formation processes for HCN and HNC that involve gas-phase mechanisms are comprehensively understood.^24−27^ Dissociative recombination reactions, as proposed by Herbst et al.,^28^
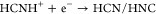
1and neutral–neutral reactions
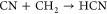
2
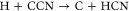
3play key roles. However, astronomical observations and astrochemical models that simulate the chemical and physical conditions over time-spans of ∼ a million years, indicate that precisely in the dense, cold, interstellar regions, reactions on ice-covered dust grains are actually pivotal in understanding HCN and HNC abundances. Lefloch et al.^18^ observed and modeled HCN-to-HNC abundance ratios of shocked regions, where an increase of the gas phase HCN abundance was detected as a consequence of grain sputtering. Similarly, the distribution of DCN along the shock necessitates the interaction between gas and grain chemistry,^29,30^ since part of the observed DCN is hypothesized to be formed in interstellar ices. In addition, HCN-to-HNC abundance ratios have been observed to be highly variable in high mass star forming regions,^16^ which has not been explained yet.

In general, astrochemical modeling codes^18,31^ only implement the surface reactions 4 and 6, leaving out the formation of HNC via reactions 5 and 7.
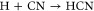
4
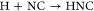
5
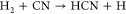
6
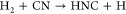
7

Reactions 4 and 5 have not been studied experimentally or theoretically on interstellar ices. On the other hand, Borget et al.^32^ experimentally studied reaction 6 in H_2_-matrix isolation experiments at 3.8 K and observed the formation of HCN as well as HCN-polymers. Previous experimental and theoretical works in the literature indicate that the associated activation energy barrier is somewhere in the range of 14.6–18.0 kJ mol^–1^, with a mild tunnelling effect (see reference^33^ and references therein).

Indeed, reactivity of radicals with molecular hydrogen has been shown to outweigh that of atomic hydrogen, despite the usual presence of activation barriers.^34−37^ Interestingly, HNC was not observed in the experiments by Borget et al.,^32^ which can be explained by the reaction 7 having a barrier of 56–70 kJ mol^–1^ in the gas phase.^33,38^ Recently, Molpeceres et al.^39^ demonstrated that hydrogenation and H-abstraction reactions on the intermediate formed after the chemisorption of carbon atoms on ammonia in water ices lead to a competition between the formation of CH_3_NH_2_ and the isomers HCN and HNC.

Up to now, astrochemical models operate under the assumption that most, if not all, species are physisorbed on the ice surface. In other words, with interactions ranging from van der Waals dispersion up to at most hydrogen bonding interaction strengths. For strongly polarizing adsorbates, however, it is known that on water ice, the most abundant interstellar ice component, the formation of hemibonded complexes may take place. This atypical type of bonding involves three electrons distributed over a pair of bonding/antibonding molecular orbitals, such that two electrons populate the former and a single one the latter, yielding a bond order of 0.5. This has indeed already been calculated for (one) specific binding site of CN on water.^40^ Furthermore, the second most abundant interstellar solid-state molecule, carbon monoxide, is known to react with CN to form the NCCO radical, with an electronic energy barrier of only 2.0 kJ mol^–1^.^41^

Despite the large band strength, HCN has not yet been directly observed on interstellar ices, with an upper limit reported by McClure et al.^42^ of 0.7–2% with respect to water. This could be either caused by the overlap of their IR signatures with those of gas-phase CO, water ice and silicates, regularly detected by JWST in these environments, or by their chemical destruction on ices.^43^ So far, the main C–N-bonded molecule in ices is OCN^–^,^42,44^ followed by the tentative detection of CH_3_CN.^45^

The destruction of HCN and HNC has been studied both theoretically and experimentally. Woon^46^ show that HCN hydrogenation can lead to CH_2_NH with an activation energy barrier of 30 kJ mol^–1^, which albeit high, may be strongly affected by quantum tunneling. This same reaction was experimentally tackled by Theule et al.,^4^ which was shown to form methylamine CH_3_NH_2_, a glycine precursor. On the other hand, Rimola et al.^40^ showed that the (CN–H_2_O)_*hemi*_ complex can undergo water assisted H-transfer reactions leading to formamide, although it has an initial energy barrier of 16.1 kJ mol^–1^. Finally, recently Boland et al.^47^ showed that HNC can rapidly react with surface OH radicals on water ices, yielding formamide after another water assisted H-transfer reaction.

Despite the importance of HCN and HNC for astrochemistry and astrobiology, their formation on interstellar ices is poorly understood on polar ices while their formation on interstellar apolar ices is entirely unexplored. In this letter we elaborate on reactions 4–6 on both H_2_O and CO molecular solids, and show that the surface formation of both species is not as trivial as it may appear at first sight.

Ices are simulated as molecular clusters of 13 to 18 units. Water ice models (of 14 and 18 molecules, (H_2_O)_14_ and (H_2_O)_18_, respectively) were extracted from the literature,^48,49^ while CO ice models (of 13 and 18 molecules, (CO)_13_ and (CO)_18_, respectively) were obtained following Ferrari et al.^50^ First, the interactions between CN radicals and each surface are examined, deriving binding energy distributions along with types of binding. Subsequently, the reactivity is detailed. M06-2X-D3(0)/def2-TZVPD has been used throughout this work,^51^ based on a benchmark at CASPT2(full valence)/aug-cc-pVTZ and CCSD(T)-F12/aug-cc-pVTZ single point energies. These are referred to here as DFT, CASPT2 and CCSD(T) for the sake of brevity. The ORCA^52^ (for DFT), Molcas^53^ (for CASPT2) and MolPro^54−56^ (for CCSD(T)) software packages were used. More information on the methods and benchmarks can be found in the Supporting Information (SI). With our benchmark (see SI) we confirm that M06-2X-D3(0) reproduces reasonably well (a) interaction energies, (b) the hemibonding interaction of CN to H_2_O and (c) the small barrier toward NCCO formation in agreement with the literature.^40,41^

*Binding Energy Distribution and Reactivity on Water Ice*. Binding energy distributions were obtained by generating 42 initial geometries, each with a randomly oriented CN molecule placed 3 Å above the water ice surface. After relaxation, only nonidentical geometries were considered, reducing the number of cases to 26 for (H_2_O)_14_ and 27 for (H_2_O)_18_ ice models. Figure 1 shows the final binding energy distribution of CN on water. The distribution is bimodal, representing both hydrogen bonding and hemibonding. With 85% the latter dominates the distribution with a mean binding energy value of 48.6 kJ mol^–1^, extending from 27.0 to 74.7 kJ mol^–1^, closely aligning with recent results by Martinez-Bachs et al.,^57^ which report a binding energy range of 21.7 to 64.8 kJ mol^–1^, and the work by Rimola et al.,^40^ who observed a binding energy of 76.6 kJ mol^–1^ in a cavity-like structure of a 33-water-molecule cluster, characterized by a high number of intermolecular interactions. This value is compatible with the high end of our distribution and the difference is likely to be produced by the different theory level, where the density functional BHandHLYP was used in combination with a double-ζ basis set for geometries and zero point energies and a triple-ζ one for energy refinement. H-bonded complexes appear at lower binding energies, with a mean value of 10.5 kJ mol^–1^. It must be noted that the wider hemibond binding energy distribution with respect to the H-bonding one is due to the combination of hemibonding (on the C atom) and H-bonding (on the N atom), as shown in the inset in Figure 1, which is naturally affected by the variety of binding sites on the cluster. Indeed, as it can be seen from Figure 1, the (H_2_O)_18_ binding energy distribution is wider than that on (H_2_O)_14_.

**Figure 1:**
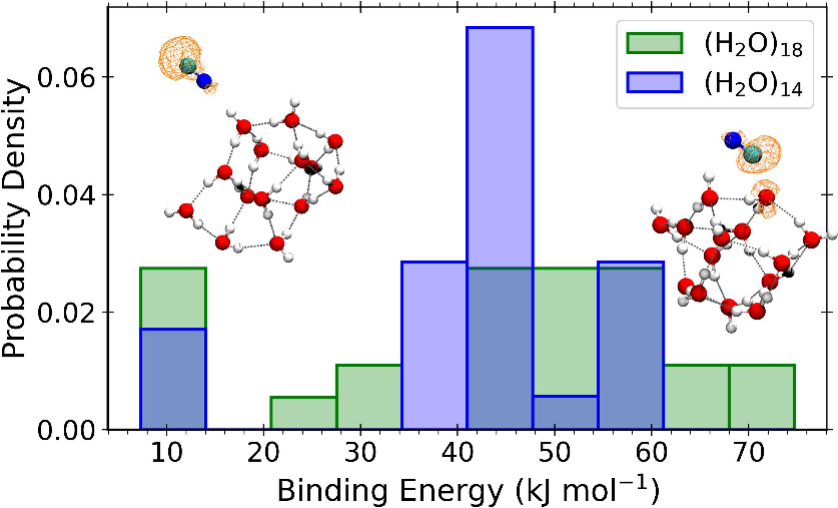
Binding energy distributions of CN on water ices. There are 26 and 27 unique sites from (H_2_O)_14_ and (H_2_O)_18_ ice models. All energies are corrected for zero point energies. The insets correspond to representative cases from the distribution, one for H-bonding and another for hemibonding, showing also the electron spin density isosurface (isovalue = 0.005 au).

The insets of this figure show also the spin density isosurfaces for both binding modes. The spin density is predominantly localized on the carbon atom for H-bonded complexes, while it is shared between CN and the surface water molecule it is hemibonded to, with a morphology similar to that of an antibonding molecular orbital, as expected for hemibonds.

Table 1 contains a summary of all the reactions studied. On water, two geometries from the binding energy distribution were taken for each binding mode, one for H-bonding and another for hemibonding. Notice we did not study the water assisted hydrogen transfer mechanism giving a precursor to formamide, since it was already discussed in the previous literature.^40^

**Table 1:** Summary of the Reactivity Energetics of CN + H/H_2_ on CO and H_2_O Ices^a^

	Hydrogen bonded on H_2_O			van der Waals bonded on CO		
	Product(s)	Barrier	*T*_*c*_	Product(s)	Barrier	*T*_*c*_
H + CN	HCN	0.0		HCN	0.0	
	HNC	–		HNC	0.0	
H_2_ + CN	HCN + H	4.7	128	HCN + H	12.0	141.2
	HNC + H	–		HNC + H	–	
						

	Hemibonded on H_2_O			Covalently bound NC–CO		
	Product(s)	Barrier	*T*_*c*_	Product(s)	Barrier	*T*_*c*_
H + CN	HCN	0.0		HCOCN	0.0	
	HNC	0.0		HCN + CO	16.7^b^	168
				HNCCO	20.2	180
H_2_ + CN	HCN + H	35.2	150	HCN + H	–	
	HNC + H	–		HNC + H	–	

a All values were ZPE-corrected, and energy units are in kJ mol^–1^ (1 kJ mol^–1^ ≃120.27 K). *T*_*c*_ are the tunneling crossover temperatures, in K. Non-reactive systems are indicated with a dash. VdW stands for van der Waals complex.

b Referenced from the asymptote.

The reactivity of H-bonded complexes is characterized by the inactivity of the N atom, as it is protected by the interaction to the surface, and the higher reactivity of the carbon atom. The formation of HCN is barrierless for reaction 4, while it sports a barrier of 4.7 kJ mol^–1^ following reaction 6 with H_2_.

On the other hand, on the hemibonded complex, we found two barrierless channels leading to HCN and HNC by simultaneously breaking the hemibond and forming the H–N or H–C bond, likely producing both products in a 1:1 ratio. We note that the product channels depend on the direction from which the H atom approaches the hemibonded complex. The reactivity of hemibonded CN with H_2_, on the other hand can only produce HCN + H, sporting a much higher barrier, of 35.2 kJ mol^–1^.

*Binding Energy Distributions and Reactivity on CO Ices*. CO ices, at difference from water ones, are not polar, and therefore one can expect reduced binding energies. We used the same method as for water ices to produce the binding energy distribution shown in Figure 2, which shows the scaled binding energy distribution of CN on CO ices. This distribution only contains van der Waals complexes with binding energies in the range of 1.9–7.0 kJ mol^–1^, and a mean value of 4.5 kJ mol^–1^, with a wider distribution from the (CO)_18_ cluster, for which a larger number of binding sites could be sampled. It must be noted that (i) M062X-D3(0) overestimates these binding energies by about a factor 2.5 as shown in the SI leading us to rescale the distribution of binding energies, and (ii) we used two highly symmetrical CO ice models for the sake of geometry optimization convergence in our calculations, see below.

**Figure 2:**
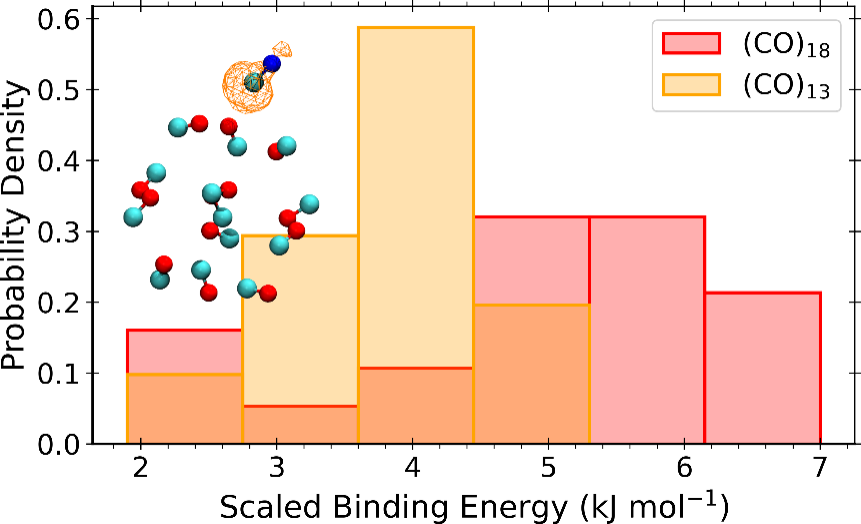
Scaled binding energy distribution of CN on CO ices. There are 12 and 22 unique instances from (CO)_13_ and (CO)_18_ ice models. All energies are corrected for zero point energies. Notice that in most cases we could not get rid of spurious imaginary frequencies, see the main text for a discussion. An example of a binding geometry is shown in the inset. This inset also contains the isosurface of the spin-density (isovalue = 0.005 au).

One geometry was chosen from the distribution to study the formation of NCCO. Indeed, this latter process happens through a low energy barrier, of 2.5 kJ mol^–1^, with respect to the van der Waals complex. This reaction has a very low energy barrier that, according to our kinetic calculations utilizing the Eckart potential model (see the SI), can be tunneled through, with a crossover temperature of 28 K (see below and the SI). This reaction is expected to take place at very low temperatures where CO is abundant on interstellar ices. Consequently, CN will remain in proximity to CO ices for a long time. Combined with the low barrier and quantum tunneling effects, this makes the formation of NCCO a highly plausible outcome.

In the following we discuss the H-addition reactivity of both binding modes, CN-CO and NCCO, with atomic and molecular hydrogen (see Table 1 for a summary).

Beginning with the van der Waals complex, it is unsurprising that the formation of HCN and HNC from reactions 4 and 5 are barrierless. In contrast, NCCO reacts with atomic hydrogen to form formyl cyanide (HCOCN) without a barrier. The formation of HCN from NCCO + H requires a barrier of 16.7 kJ mol^–1^ (from the asymptote) in which the NC–CO is simultaneously broken. Interestingly, IRC calculations from NCCO + H → HCN + CO toward reactants lead also barrierlessly to the formation of HCOCN. Finally, the reaction of atomic hydrogen on the N atom of NCCO leads to the HNCCO radical, which features a high energy barrier of 20.2 kJ mol^–1^.

Concerning reactivity with H_2_, it exclusively applies to the van der Waals complex, resulting in the formation of HCN with a barrier of 12.0 kJ mol^–1^.

We want to highlight the technical difficulties while working with CO ices as a consequences of the van der Waals CO–CO interactions. This translates into extreme difficulty to converge geometry optimizations. As a result, we have allowed up to 2–9 imaginary frequencies, smaller than ∼50 cm^–1^, which are not included in the zero point energies. The change of including all imaginary frequencies in the ZPE as real values has a limited effect ≤1 kJ mol^–1^ on average.

*Astrochemical Implications*. Desorption rates depend exponentially on binding energy values, and as a result, the desorption from water is a much less likely process than from CO. The longer residence times on water dramatically increases the chances of the reactions between (CN–H_2_O)_*hemi*_ and atomic or molecular hydrogen. The first reaction efficiently yields both HCN and HNC, depending solely on the direction from which the H atom approaches, while the second with H_2_ is much less likely given the high energy barrier and consequent low rate constants (see Figure 3). On the contrary, the few CN radicals on water on the H-bonding mode will efficiently form only HCN, given that the reaction of (CN–H_2_O)_*H*−*bond*_ with H is barrierless and that with H_2_ has a low energy barrier and experiences a strong quantum tunnel effect, with rate constants around 10^8^–10^9^ s^–1^ between 10 and 30 K.

**Figure 3:**
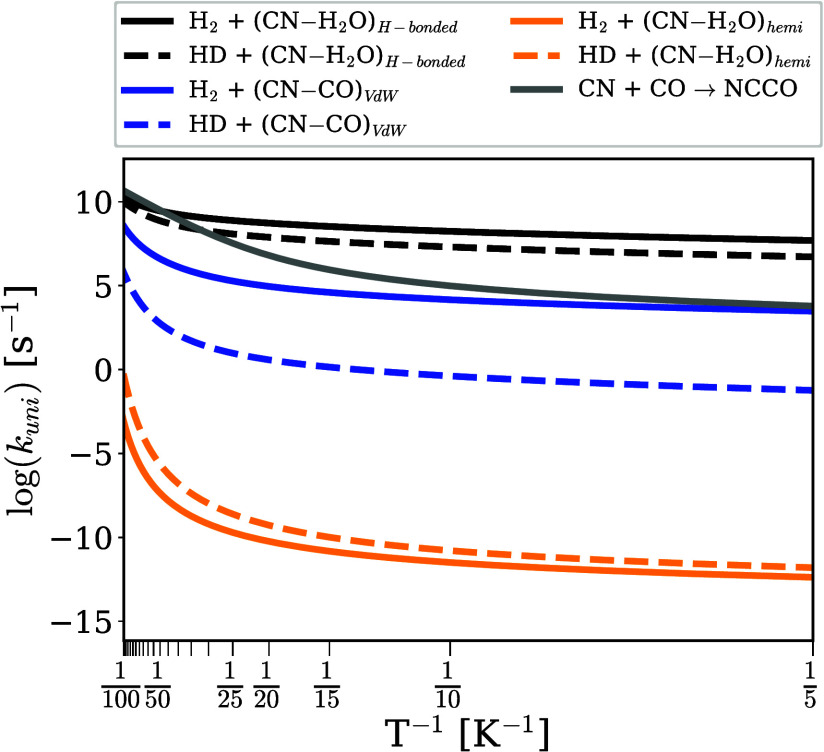
Rate constants for NCCO formation and H_2_ + CN in three binding modes (see SI for further details). The subscripts “VdW”, “H-bonded”, and “hemi” stand for van der Waals, hydrogen bonded, and hemibonded, respectively.

In colder and denser regions of the ISM, CO accumulates on the icy surfaces and most of the hydrogen exists in molecular form. The H_2_ + (CN–CO)_*VdW*_ reaction is likely accessible thanks to the mild barrier height (12.0 kJ mol^–1^) and its strong quantum tunneling effect, with rate constants around 10^4^–10^5^ s^–1^ between 10 and 30 K, which would form only HCN. Note that the barrier height on CO is close to the gas phase value (13.3 kJ mol^–1^, as reported by Ju et al.^58^). However, one should not overlook the - albeit limited - presence of atomic hydrogen, with fractional abundances around ∼10^–5^–10^–2^ relative to the global proton abundance.^59^ This may still lead to the production of both HCN and HNC at intermediate visual extinction regions where CO just starts to freeze out and atomic hydrogen is still present.

Finally, NCCO can be formed on the van der Waals complex binding sites. This reaction, with a very low energy barrier of just 2.5 kJ mol^–1^, has also been found to experience quantum tunneling, rendering the reaction very fast, with rate constants in the 10^5^–10^8^ s^–1^ range, between 10 and 30 K, faster than H_2_ + (CN–CO)_*VdW*_. In addition, besides hydrogenation with atomic H, another competition channel in detriment of NCCO formation would be the desorption of CN from CO ices. Under the assumptions mentioned above for NCCO formation, and describing the desorption rate constant of CN constant following Tielens and Allamandola^60^ (*k* = ν exp(−*E*_*bind*_/*Tk*_*B*_) with  and *N*_*s*_ = 10^15^ m^–2^), NCCO formation goes from being in competition with desorption at 30 K, to completely dominate their ratio at 10 K.

Once NCCO is formed, it can barrierlessly react with atomic hydrogen to form HCOCN. This molecule itself can undergo further hydrogenation. The activation energy barriers depending on the formed product. In the gas phase, and not including ZPE effects, these barriers at M062X-D3(0)/def2-VTZPD level are 17.4 kJ mol^–1^ (H_2_COCN), 19.0 kJ mol^–1^ (HCO(CH)N), 24.7 kJ mol^–1^ (HCOC(NH)), 31.9 kJ mol^–1^ (HC(OH)CN) and 40.2 kJ mol^–1^ (NCCO + H_2_). The lowest barriers are toward H_2_COCN and HCO(CH)N, both with high crossover temperatures of 214 and 177 K, respectively. If these two intermediates form, they could further yield hydroxyacetonitrile (OHCH_2_CN), recently detected in the interstellar medium by Zeng et al.,^61^ and 2-iminoacetaldehyde (CHOCHNH) through further barrierless H-additions, both with great prebiotic importance.

Given that barrierless reactions will remain barrierless if hydrogen is substituted by deuterium, we further investigated the effects of deuteration only for the most important reactions involving a reaction energy barrier.

For this discussion, we present the rate constants in Figure 3, with the temperature-dependent kinetic isotope effects (KIEs) detailed in the Supporting Information (SI). As shown in Figure 3, the reaction with HD + CN → DCN + H is generally slower than H_2_ + CN → HCN + H, except for the hemibonded CN radical. This reaction exhibits a KIE < 1 in the 10—30 K range, meaning that the formation of DCN is (up to 10 times) faster than the formation of HCN. This is a consequence of the zero-point energy correction lowering the energy barrier. However, given its already very slow rate constant (under 10^–10^ s^–1^), this deuteration channel is not expected to be significant.

Furthermore, the reaction of hydrogen bonded CN, with a KIE of 6–9 in the same temperature range, is also of little importance because H-bonding is not the dominant binding mode of CN on water ices. On the other hand, H_2_/HD + CN → HCN/DCN + H of van der Waals bound CN competes with NCCO formation at slightly elevated temperatures. The formation of HCN is likely still relevant as a consequence of the high molecular hydrogen abundance in molecular clouds. However, with a KIE of about 10^4^–10^5^, the rate constant for DCN formation is reduced to a few s^–1^, rendering this reaction negligible.

In conclusion, the most important sources of DCN and DNC are the atom addition reactions (D + CN), which depend directly on the availability of deuterium atoms on the surface, i.e., the gas-phase D/H ratio and the D accretion rates.

In the following paragraph, we propose some recommendations to astrochemical modelers to include our conclusions, deeply rooted in physical chemical insight, in their work:

- On water-ice surfaces, the dominant binding mode is hemibonded, with a mean binding energy of 48.6 kJ mol^–1^.
  - This leads on average to a 1:1 mixture of HCN and HNC by the reaction H + CN
  - On the other hand, the reaction H_2_ + CN does not play an important role given the high energy barrier.
  - The formation of HCN and HNC from H + CN is in competition with formamide formation^40^
- The smaller amount of H-bonded binding modes (10.5 kJ mol^–1^) on water ice only leads to HCN formation, for both H + CN and H_2_ + CN reactions
- In deeper regions of the molecular cloud where CN will primarily interact with CO, the dominant interaction is weak, of 4.5 kJ mol^–1^.
  - The reaction H + CN can lead to both HCN and HNC in an expected 1:1 ratio
  - The reaction H_2_ + CN is also at work via quantum tunnelling producing only HCN
  - We expect a competition between the reactions H/H_2_ + CN and the formation of NCCO
  - NCCO can be further hydrogenated to HCOCN in a barrierless fashion

Overall, quantum tunneling plays a central role and we expect more HCN to be formed than HNC, with a small fraction of CN radicals converted into formamide on H_2_O ices, but a significant fraction of CN radicals converted to HCOCN on CO ices. This contributes to a further understanding of the lack of HCN and HNC detections in ices with JWST.

Summarizing, taking into account the dominant binding mode on water and carbon monoxide, the reaction between H and CN will lead to both HCN and HNC, whereas the reaction with H_2_ will only produce HCN. Furthermore, both product channels are in competition with the formation of NH_2_CHO on water and HCOCN on carbon monoxide.

Upcoming work will implement our novel formation pathways, along with known destruction pathways^4,32,40,46,47^ into an astrochemical model to study (a) how long-lived HCN and HNC are on interstellar ices, (b) to explore the HCN/HNC ratio and (c) the DCN/HCN ratio across physical conditions.

With this work we show that it is of pivotal importance to build astrochemistry on the strong foundations of accurate quantum chemical calculations and physical chemical studies for reactions and species that are known to be important, not only as tracers in the ISM, but also as precursors for complex, prebiotic species.

## Supporting Information Available

Functional benchmark, exploration of the CN–H_2_O hemibond and NCCO radical formation, kinetics and binding energy tables (PDF)
