# Supplementary material for: The Complex (Organic) Puzzle of the Formation of Hydrogen Cyanide and Isocyanide on Interstellar Ice Analogues
Source: J Phys Chem Lett. 2024 Jul 25;15(30):7799–805. doi: 10.1021/acs.jpclett.4c01537 (PMC11299174; doi:10.1021/acs.jpclett.4c01537)
Supplement: Supplementary file 1 — jz4c01537_si_001.pdf [file jz4c01537_si_001.pdf]

Suporting Information for "The Complex (Organic) Puzzle of the Formation of Hydrogen Cyanide and Isocyanide on Interstellar Ice Analogs"

Joan Enrique-Romero[*,†] and Thanja Lamberts[*,†,‡]

†*Leiden Institute of Chemistry, Gorlaeus Laboratories, Leiden University, PO Box 9502, 2300 RA Leiden, The Netherlands*

‡*Leiden Observatory, Leiden University, P.O. Box 9513, 2300 RA Leiden, The Netherlands*

E-mail: j.enrique.romero@lic.leidenuniv.nl; a.l.m.lamberts@lic.leidenuniv.nl

Contents

1 CN-Water H-bonding interaction S2

2 CN-CO Van der Waals interaction S4

3 CN-water hemibond and NCCO radical formation S6

4 Reactivity on hemibonded CN-H2O S12

5 Reactivity of NC···CO and NCCO S13

6 Gas-phase reaction $CN + H_2 \rightarrow HCN + H$ S16

7 NCCO formation on the surface S17

8 Kinetics calculations S18

9 Binding energy tables S20

References S21

1 CN-Water H-bonding interaction

The H-bonding interaction of CN to a water trimer (Figure 1) was used to study the broader performance of different density functionals compared to CCSD(T)-F12/aug-cc-pVTZ (Table 1). Indeed M062X-D3 performs well.

Notice that the average binding energy value for the H-bonded complex (obtained in the distribution in the main text), is of 10.5 kJ mol^{-1}, which deviates from the values reported by Wakelam et al.[1] (20.8–24.9 kJ mol−1), while it is closer to the value of 13.3 kJ mol−1, used in, e.g., Garrod and Herbst[2], Belloche et al.[3].

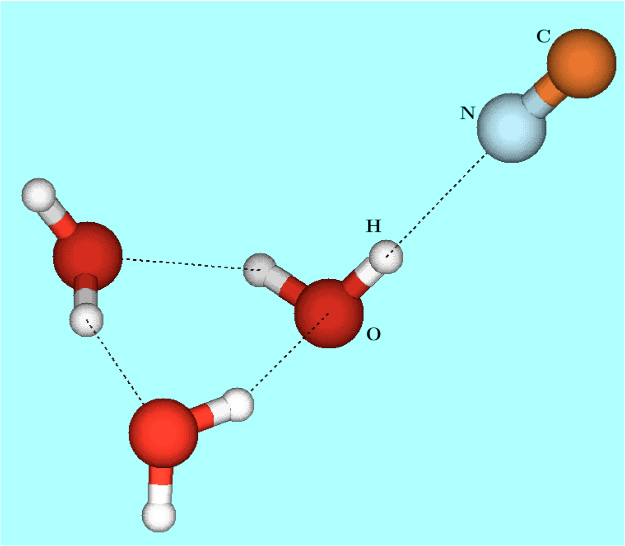

Figure 1: Geometry used for the benchmark of CN$-$H$_2$O H-bonding. The N$-$O distance is 2.147 Å.

Table 1: Single point energy benchmark for H-bonding interaction between CN and a water trimer. Bare energies in Hartree. ΔE^{inter}: electronic interaction energy, with no further corrections. Δ: interaction energy difference between DFT and coupled cluster. MUE(%): Mean unsigned error. All DFT calculations are combined with a def2-TZVP basis set, and CCSD(T)-F12 with AUG-CC-pVTZ. Initial geometries were generated at BHLYP-D4/def2-VTZP level.

	$(H_2O)_3$	CN	Complex	ΔE^{inter}	Δ	MUE(%)
CCSD(T)-F12	-229.1216786	-92.59348082	-321.7188067	-9.6		
B3LYP-D4	-229.3089078	-92.70126912	-322.0142176	-10.6	1.0	10.8
BHLYP-D4	-229.2902105	-92.69032847	-321.9853373	-12.6	3.0	31.6
M062X-D3	-229.3089002	-92.71165264	-322.0249415	-11.5	1.9	20.3
MPWB1K-D3(BJ)	-229.2939215	-92.69458445	-321.9923908	-10.2	0.6	6.5
PBE-D4	-229.1618771	-92.64136419	-321.811433	-21.5	11.9	124.6
PW6B95-D4	-229.6515571	-92.86166263	-322.5169662	-9.8	0.3	2.7
TPSSH-D3(BJ)	-229.3971738	-92.75281972	-322.1540691	-10.7	1.1	11.7
wB97m-D3(BJ)	-229.4489389	-92.77422575	-322.2272197	-10.6	1.1	11.2
wB97m-V	-229.3190108	-92.718747	-322.0416742	-10.3	0.7	7.4
wB97x-D4	-229.4508461	-92.76703654	-322.2217044	-10.0	0.5	4.8

2 CN-CO Van der Waals interaction

Table 2 lists the CN-CO Van der Waals interaction benchmark data. While highly correlated post-HF methods indicate interaction energies of around 3 kJ mol^{-1}, DFT and MP2 consistently show interaction energies larger than about 6 kJ mol^{-1}, with the best performing functionals being MPWB1K and wB97m-V. Despite of this we continue our study with M062X-D3(0) to be as systematic as possible. At the same time, however, we acknowledge that the resulting binding energies need to be scaled (e.g.,.[4] To obtain the scaling factor we run an additional set of four M062X-D3(0) optimizations of an adsorbed CN radical on a cluster of 3 CO molecules, using CCSD(T)-F12/aug-cc-VTZ as a reference, see Table 3. An average M062X-D3(0)/CCSD(T)-F12 ratio of 2.5 is derived using the geometries in Figure 2.

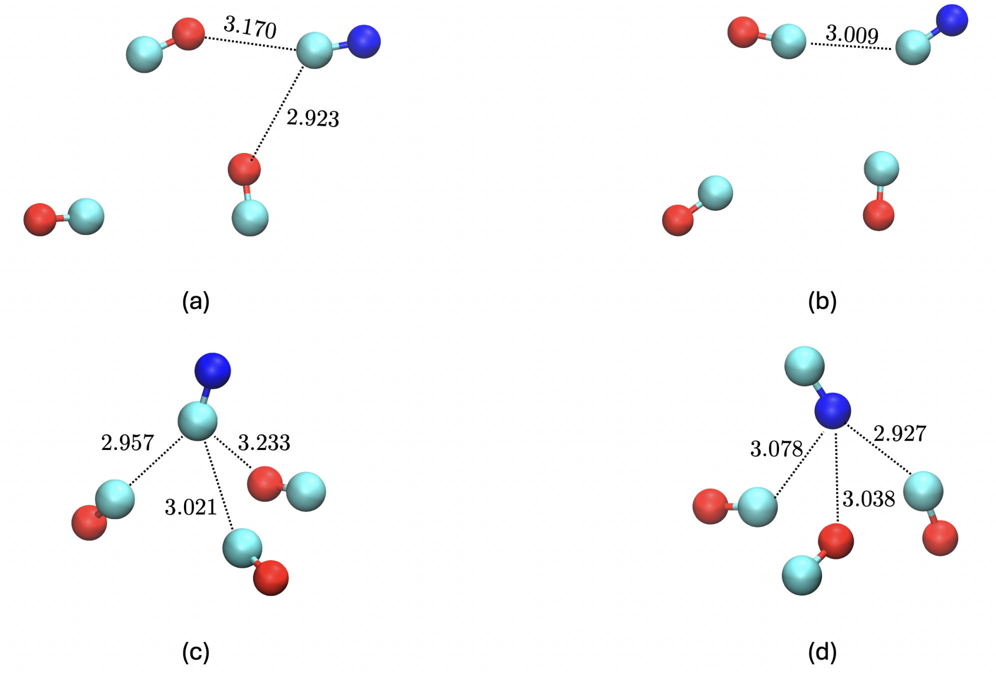

Figure 2: Geometries used to obtain the scaling factor for CN$\cdots$$(CO)_3$ Van der Waals complexes. Distances in Å.

Finally, we would like to point out that we decided not to include basis set superposition error corrections to our binding energies because of the impossibility to converge some of the self-consistent-field calculations with ghost atoms when using the counterpoise method.

Table 2: CN-CO Van der Waals interaction benchmark. Raw energies in Hartree, relative ones in kJ mol^{-1}. All DFT methods and the DLPNO-CCSD(T) are combined with a def2-VTZPD basis set. All calculations where run with ORCA except for CCSD(T)-F12 and the AVAS-PT2 which where run with Molpro, both combined with the aug-cc-pvtz basis set. In the latter the active space is composed solely of the 2s and 2p orbitals of C and N in the CN radical. MP2 calculations where run with ORCA and combined with the aug-cc-pvtz basis set.

Method	CN	CO	Complex	dE
CCSD(T)-F12	-92.59407838	-113.1926509	-205.7878448	-2.9
DLPNO-CCSD(T)	-92.56883346	-113.1592983	-205.7291481	-2.7
QCISD(T)	-92.57279036	-113.1624143	-205.7366469	-3.8
AVAS-PT2*	-205.6870944	–	-205.6882439	-3.0
MP2	-92.5190052	-113.1416513	-205.6636446	-7.8
B3LYP-D4	-92.70183158	-113.3112616	-206.0159098	-7.4
BHLYP-D4	-92.69042827	-113.3054933	-205.9984022	-6.5
BLYP-D4	-92.74078299	-113.3500473	-206.0943719	-9.3
M052X-D3(0)	-92.73100664	-113.3488206	-206.0827222	-7.6
M062X-D3(0)	-92.71193267	-113.322595	-206.0376386	-8.2
M08HX-D3(0)	-92.70891514	-113.3220003	-206.0333395	-6.4
MPWB1K-D3(BJ)	-92.69478605	-113.310365	-206.007566	-6.3
PBE-D4	-92.64229271	-113.2346347	-205.8810597	-10.8
PW6B95-D4	-92.86210798	-113.4949627	-206.3597963	-7.2
R2SCAN-D3(BJ)	-92.69744015	-113.2988874	-205.9989719	-6.9
RSCAN-D3(BJ)	-92.72768196	-113.334812	-206.065644	-8.3
TPSSH-D3(BJ)	-92.75345783	-113.360159	-206.1164464	-7.4
wB97m-D3(BJ)	-92.77472983	-113.3988349	-206.1760738	-6.6
wB97m-V	-92.71925522	-113.3390431	-206.0607261	-6.4
wB97X-D4	-92.76759872	-113.3859344	-206.1561185	-6.8

* Instead of starting from the asymptote, the NC−CO distance was set to 10 Å.

Table 3: Binding energy ratios of Van der Waals compelxes of CN on a $(CO)_3$ ice model. Raw energies in Hartree.

	Raw Energies		Binding energy ratio
	CCSD(T)-F12	M062X-D3(0)	
CN radical	-92.59407838	-92.71193215	
$(CO)_3$	-339.5796071	-339.9692166	
Complex 1	-432.1750653	-432.6835522	1.7
Complex 2	-432.1755474	-432.6861347	2.7
Complex 3	-432.1757032	-432.6867103	2.8
Complex 4	-432.1759274	-432.6874884	2.8
		**Average**	**2.5**

It must be noted that for the successful cases the corrections were lower than 1 kJ mol^{-1}, (0.6 kJ mol^{-1} on average for CO ices), and that we are already using a large basis set of triple-ζ quality. These two effects, are not as important in our Van der Waals binding energy results on CO given the overestimation of these energies by the chosen functional and the consequent rescale.

3 CN-water hemibond and NCCO radical formation

The interaction of CN on H_2O or CO clusters is not as straightforward as one might think, since it can form hemibonded complexes with H_2O and it can react with CO to form the NCCO radical. Therefore, we investigate the potential energy surfaces of their interactions. In order to do so, first, a benchmark on smaller models is performed to determine an accurate density functional to properly describe these interactions later on with larger clusters. This has been done for both $(\mathrm{CN}\cdots\mathrm{H_2O})_{hemi}$ (Table 4) and NCCO (Table 5) using CASPT2 single points with an active space covering all valence electrons and orbitals. The initial geometries for these single points were obtained by generating an array of geometries where the distance between the two bonding atoms (NC$-$OH$_2$ and NC$-$CO, respectively) is varied, keeping all other geometrical parameters constrained to the optimized structure of the complex as obtained at the BHandHLYP/def2-VTZPD level.

For both $(\mathrm{CN}\cdots\mathrm{H_2O})_{hemi}$ and NCCO, the best performing functional is M062X-D3(0)/def2-TZVPD, see Tab. 4 and 5. It properly predicts the minimum energy bond distance $(\mathrm{CN}\cdots\mathrm{H_2O})_{hemi}$ and is the only functional to predict an energy barrier for the $\mathrm{CN} + \mathrm{CO} \rightarrow$ NCCO reaction. We checked that it is important to include a diffuse function in the basis set for obtaining a good agreement with the CASPT2 full valence values.

Fully optimized stationary points of the CN+CO $\rightarrow$ NCCO reaction at M062X-D3(0)/def2-VTZPD level are depicted in Figure 4. The obtained activation and reaction energies including ZPE corrections are is 2.2 kJ mol^{-1} and -122.7 kJ mol^{-1}, in good agreement with the 2.1

kJ mol^{-1} and -110.0 kJ mol^{-1} found by Yu et al.[5] at CCSD(T)/6-311+G(2df)//QCISD/6-311G(d)+ZPVE level.

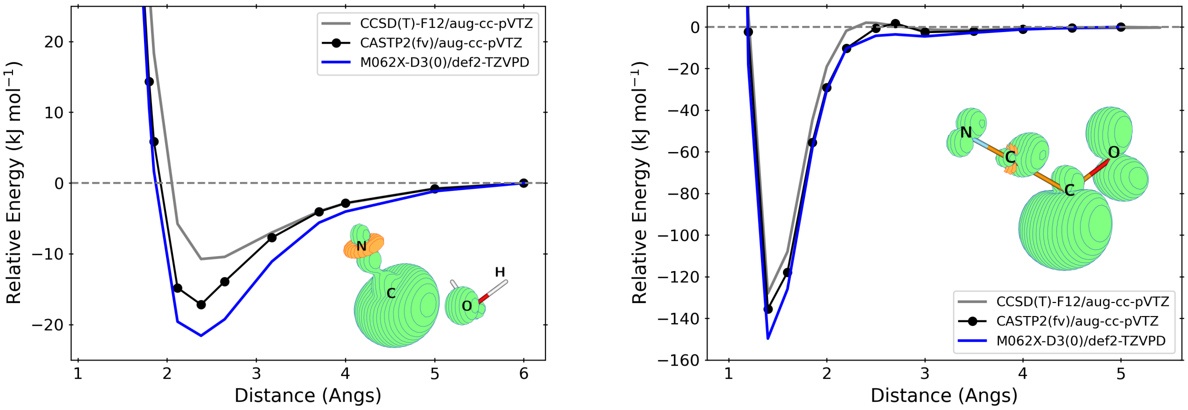

Figure 3: Potential energy surfaces for the formation of the CN−H_2O hemibond (left panel) and the NCCO radical (right panel). The insets correspond to the spin densities in the DFT interaction wells using 0.01 isosurfaces.

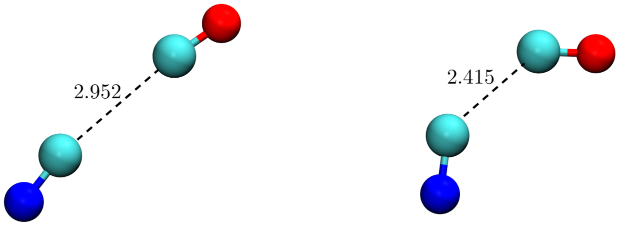

Figure 4: PES stationary points for NCCO formation from CN and a sigle CO molecule. Distances in Å.

Furthermore, as a final confirmation, also the spin density along the PES, at key distances for the formation of the CN−H_2O hemibond and the NCCO radical, compare well between DFT and CASPT2(fv), see Figure 5.

We further confirm that the observed barrier towards NCCO formation is physically sound and not just an artifact due to, e.g., higher electronic states mixing into the CASPT2 and CCSDT-F12 solutions. We run an extended multi-state XMS-CASTP2(full valence)/aug-cc-PVTZ (MS-CASPT2) calculation exploring the first three electronic states of the NCCO

Table 4: Comparison of CN−H_2O_{hemi} interaction curves with different functionals C−O distance in Å, bare energies (upper table) in Hartree, relative energies (lower table) in kJ mol^{-1}. All DFT calculations were combined with the def2-TZVPD basis set. CASPT2(fv) indicates a CASPT2/AUG-CC-pVTZ//CASSCF/AUG-CC-pVTZ calculation where the active space expands the whole valence space of the complex (17 electrons and 14 orbitals). The initial geometries were generated by manually displacing the minimum calculated at BHLYP-D4/def2-TZVP level.

Distance	CASPT2(fv)	CCSD(T)-F12	B3LYP-D4	BHLYP-D4	M062X-D3	MPWB1K-D3(BJ)	PBE-D4	PW6B96-D4	TPSSH-D3(BJ)	wB97M-V	wB97X-D3	wB97X-D4
1.164	-168.7397591	-168.8361514	-169.0122703	-168.9905392	-169.0171826	-169.0034586	-168.9121665	-169.2890624	-169.0906288	-169.0313075	-169.0287219	-169.1174935
1.270	-168.7915698	-168.8838435	-169.0581809	-169.0369647	-169.0642124	-169.0487997	-168.9578327	-169.3344743	-169.1374939	-169.0785323	-169.0763867	-169.1665572
1.376	-168.8155836	-168.908542	-169.0814355	-169.060939	-169.0880953	-169.0717341	-168.9805291	-169.3574337	-169.1619481	-169.103437	-169.1014476	-169.1926416
1.482	-168.8316296	-168.9244347	-169.0960478	-169.0764677	-169.1031466	-169.0863596	-168.9940682	-169.3720002	-169.1776087	-169.1194508	-169.117913	-169.2094686
1.588	-168.8451468	-168.9378628	-169.1085871	-169.0898009	-169.1163673	-169.0989166	-169.0051927	-169.3845377	-169.1906071	-169.1325744	-169.1317312	-169.2232566
1.693	-168.8569815	-168.9496021	-169.1199002	-169.1015682	-169.1279198	-169.1099811	-169.0152168	-169.3957082	-169.2019096	-169.143776	-169.1435867	-169.2349889
1.799	-168.8655188	-168.9588313	-169.1290681	-169.1107688	-169.1368685	-169.1185628	-169.0234929	-169.4045739	-169.2109118	-169.1524199	-169.1527701	-169.2440668
1.852	-168.8687426	-168.9623734	-169.1326308	-169.1142327	-169.1402458	-169.1217706	-169.0267628	-169.4079568	-169.2144169	-169.155659	-169.1562532	-169.2474606
2.117	-168.8766255	-168.9715467	-169.1414247	-169.1222196	-169.1483465	-169.1289295	-169.0351063	-169.4160154	-169.2229991	-169.1628544	-169.1644142	-169.2549466
2.381	-168.8775251	-168.9734414	-169.1417722	-169.1222531	-169.1491057	-169.1284994	-169.0355234	-169.416041	-169.2231721	-169.1621134	-169.1642676	-169.2542995
2.646	-168.8762843	-168.973326	-169.1396056	-169.1204673	-169.1482179	-169.126474	-169.0332908	-169.4139182	-169.2209	-169.1596562	-169.1619722	-169.2519041
3.175	-168.8739264	-168.9720173	-169.1353493	-169.117328	-169.1451109	-169.1233566	-169.0283786	-169.410154	-169.2166644	-169.1559406	-169.1579839	-169.2483147
3.704	-168.8725318	-168.9708745	-169.1330902	-169.1155242	-169.1430196	-169.1216937	-169.0253354	-169.4081691	-169.2144699	-169.1541363	-169.1560221	-169.2465184
4.000	-168.8720637	-168.970438	-169.1324521	-169.1149323	-169.1424234	-169.1211859	-169.024344	-169.4075984	-169.2138124	-169.1535983	-169.155459	-169.2459621
5.000	-168.8712859	-168.969648	-169.1315793	-169.1139718	-169.1413261	-169.1202651	-169.0228366	-169.4066376	-169.212773	-169.152709	-169.1545206	-169.2450594
6.000	-168.8709855	-168.969352	-169.1312895	-169.1136125	-169.1408866	-169.1198901	-169.0224115	-169.4062726	-169.212429	-169.1523643	-169.1541871	-169.2447442

Distance	CASPT2(fv)	CCSD(T)-F12	B3LYP-D4	BHLYP-D4	M062X-D3	MPWB1K-D3(BJ)	PBE-D4	PW6B96-D4	TPSSH-D3(BJ)	wB97M-V	wB97X-D3	wB97X-D4
1.164	344.5	349.7	312.5	323.1	324.8	305.7	289.4	307.7	319.8	317.8	329.4	334.1
1.270	208.5	224.5	191.9	201.2	201.3	186.6	169.6	188.5	196.7	193.8	204.3	205.3
1.376	145.5	159.7	130.9	138.3	138.6	126.4	110.0	128.2	132.5	128.5	138.5	136.8
1.482	103.3	117.9	92.5	97.5	99.1	88.0	74.4	90.0	91.4	86.4	95.2	92.6
1.588	67.8	82.7	59.6	62.5	64.4	55.1	45.2	57.1	57.3	52.0	59.0	56.4
1.693	36.8	51.9	29.9	31.6	34.0	26.0	18.9	27.7	27.6	22.5	27.8	25.6
1.799	14.4	27.6	5.8	7.5	10.5	3.5	-2.8	4.5	4.0	-0.1	3.7	1.8
1.852	5.9	18.3	-3.5	-1.6	1.7	-4.9	-11.4	-4.4	-5.2	-8.7	-5.4	-7.1
2.117	-14.8	-5.8	-26.6	-22.6	-19.6	-23.7	-33.3	-25.6	-27.8	-27.5	-26.9	-26.8
2.381	-17.2	-10.7	-27.5	-22.7	-21.6	-22.6	-34.4	-25.6	-28.2	-25.6	-26.5	-25.1
2.646	-13.9	-10.4	-21.8	-18.0	-19.2	-17.3	-28.6	-20.1	-22.2	-19.1	-20.4	-18.8
3.175	-7.7	-7.0	-10.7	-9.8	-11.1	-9.1	-15.7	-10.2	-11.1	-9.4	-10.0	-9.4
3.704	-4.1	-4.0	-4.7	-5.0	-5.6	-4.7	-7.7	-5.0	-5.4	-4.7	-4.8	-4.7
4.000	-2.8	-2.9	-3.1	-3.5	-4.0	-3.4	-5.1	-3.5	-3.6	-3.2	-3.3	-3.2
5.000	-0.8	-0.8	-0.8	-0.9	-1.2	-1.0	-1.1	-1.0	-0.9	-0.9	-0.9	-0.8
6.000	0.0	0.0	0.0	0.0	0.0	0.0	0.0	0.0	0.0	0.0	0.0	0.0

Table 5: Comparison of NCCO formation curves with different functionals. C−C distance in Å, bare energies (upper table) in Hartree, relative energies (lower table) in kJ mol^{-1}. All DFT calculations were combined with the def2-TZVPD basis set. CASPT2(fv) indicates a CASPT2/AUG-CC-pVTZ//CASSCF/AUG-CC-pVTZ calculation where the active space expands the whole valence space of the complex (19 electrons and 15 orbitals). The initial geometries were generated by manually displacing the minimum calculated at BHLYP-D4/def2-TZVP level. We encountered problems with some CASPT2 calculations, which could not manage to converge and are left in blank.

Distance	CASPT2(fv)	CCSD(T)-F12	B3LYP-D4	BHLYP-D4	M062X-D3	MPWB1K-D3(BJ)	PBE-D4	PW6B96-D4	TPSSH-D3BJ	wB97M-V	wB97X-D4
1	-205.4513589	-205.5477637	-205.8023112	-205.7800574	-205.8041133	-205.796381	-205.6742249	-206.149643	-205.903862	-205.8332746	-205.9269191
1.2	-205.6831918	-205.7787434	-206.0265524	-206.0072427	-206.0349279	-206.0212566	-205.8984122	-206.3723793	-206.1278821	-206.0633163	-206.1597693
1.4	-205.7339193	-205.8301912	-206.0723468	-206.0542385	-206.085144	-206.0667489	-205.9438211	-206.4171082	-206.1737136	-206.1128903	-206.2112295
1.6	-205.7271965	-205.822566	-206.0617132	-206.0422832	-206.0760875	-206.0541379	-205.9334996	-206.4053733	-206.163403	-206.1039542	-206.2026982
1.854	-205.7034466	-205.7984792	-206.0359667	-206.0132703	-206.0502386	-206.0247748	-205.9085774	-206.378342	-206.1382024	-206.0781669	-206.1760221
2	-205.6933789	-205.7886954	-206.0254723	-206.0019164	-206.0397256	-206.0130056	-205.8977786	-206.3674597	-206.1277359	-206.0671947	-206.1648815
2.2	-205.6862191	-205.7821319	-206.0169574	-205.9937779	-206.0320647	-206.0044628	-205.8880954	-206.3590802	-206.1191394	-206.059023	-206.1560761
2.4	-205.6825511	-205.7806542	-206.0129221	-205.9906676	-206.029604	-206.0009546	-205.8826872	-206.3553737	-206.1147413	-206.0557958	-206.1521561
2.5	-205.6816598	-205.780708	-206.0117768	-205.989994	-206.0294826	-206.0000672	-205.8809343	-206.3543537	-206.1134281	-206.0550272	-206.1511361
2.7	-205.6829185	-205.7812171	-206.0103023	-205.9893632	-206.0297339	-205.9991509	-205.8784691	-206.35314	-206.1117243	-206.0541875	-206.1499905
2.8		-205.7814777	-206.0097986	-205.9892084	-206.0298098	-205.9988739	-205.8775711	-206.3527291	-206.1111606	-206.0539401	-206.1496527
3	-205.6832725	-205.7818444	-206.0090508	-205.988994	-206.029846	-205.9985446	-205.8761965	-206.352159	-206.1103785	-206.0535795	-206.1491958
3.1		-205.7819437	-206.0087696	-205.9889032	-206.0297586	-205.9984234	-205.8756754	-206.3519439	-206.1101168	-206.0534362	-206.1490258
3.3		-205.7820156	-206.0083256	-205.988724	-206.0294709	-205.9982481	-205.8748579	-206.3516287	-206.1097309	-206.0531853	-206.1487424
3.5	-205.683054	-205.7819855	-206.0080001	-205.9885506	-206.0291686	-205.9980523	-205.8742758	-206.3513438	-206.1094603	-206.0529851	-206.1485102
3.7		-205.7819014	-206.0077665	-205.9884001	-206.0289129	-205.9979893	-205.8738712	-206.3511918	-206.1092696	-206.0528305	-206.1483292
4	-205.6827034	-205.7817509	-206.0075103	-205.9882103	-206.0285699	-205.9977682	-205.873429	-206.3509308	-206.1089922	-206.0526576	-206.1481081
4.5	-205.6824597	-205.7815522	-206.0073118	-205.988042	-206.028266	-205.9975935	-205.8730518	-206.350705	-206.1087031	-206.0524971	-206.1479102
5	-205.6823119	-205.7814353	-206.0071946	-205.9879447	-206.0281173	-205.9974614	-205.8728313	-206.3505354	-206.1084948	-206.0523798	-206.1477895

Distance	CASPT2(fv)	CCSD(T)F12/AVTZ	B3LYP-D4	BHLYP-D4	M062X-D3	MPWB1K-D3(BJ)	PBE-D4	PW6B96-D4	TPSSH-D3BJ	wB97M-V	wB97X-D4
1	606.4	613.5	537.9	545.8	588.1	527.9	521.4	527.4	537.3	575.3	579.9
1.2	-2.3	7.1	-50.8	-50.7	-17.9	-62.5	-67.2	-57.4	-50.9	-28.7	-31.5
1.4	-135.5	-128.0	-171.1	-174.1	-149.7	-181.9	-186.4	-174.8	-171.2	-158.9	-166.6
1.6	-117.8	-108.0	-143.1	-142.7	-125.9	-148.8	-159.3	-144.0	-144.2	-135.4	-144.2
1.854	-55.5	-44.7	-75.5	-66.5	-58.1	-71.7	-93.9	-73.0	-78.0	-67.7	-74.1
2	-29.1	-19.1	-48.0	-36.7	-30.5	-40.8	-65.5	-44.4	-50.5	-38.9	-44.9
2.2	-10.3	-1.8	-25.6	-15.3	-10.4	-18.4	-40.1	-22.4	-27.9	-17.4	-21.8
2.4	-0.6	2.1	-15.0	-7.1	-3.9	-9.2	-25.9	-12.7	-16.4	-9.0	-11.5
2.5	1.7	1.9	-12.0	-5.4	-3.6	-6.8	-21.3	-10.0	-13.0	-7.0	-8.8
2.7	-1.6	0.6	-8.2	-3.7	-4.2	-4.4	-14.8	-6.8	-8.5	-4.7	-5.8
2.8		-0.1	-6.8	-3.3	-4.4	-3.7	-12.4	-5.8	-7.0	-4.1	-4.9
3	-2.5	-1.1	-4.9	-2.8	-4.5	-2.8	-8.8	-4.3	-4.9	-3.1	-3.7
3.1		-1.3	-4.1	-2.5	-4.3	-2.5	-7.5	-3.7	-4.3	-2.8	-3.2
3.3		-1.5	-3.0	-2.0	-3.6	-2.1	-5.3	-2.9	-3.2	-2.1	-2.5
3.5	-1.9	-1.4	-2.1	-1.6	-2.8	-1.6	-3.8	-2.1	-2.5	-1.6	-1.9
3.7		-1.2	-1.5	-1.2	-2.1	-1.4	-2.7	-1.7	-2.0	-1.2	-1.4
4	-1.0	-0.8	-0.8	-0.7	-1.2	-0.8	-1.6	-1.0	-1.3	-0.7	-0.8
4.5	-0.4	-0.3	-0.3	-0.3	-0.4	-0.3	-0.6	-0.4	-0.5	-0.3	-0.3
5	0.0	0.0	0.0	0.0	0.0	0.0	0.0	0.0	0.0	0.0	0.0

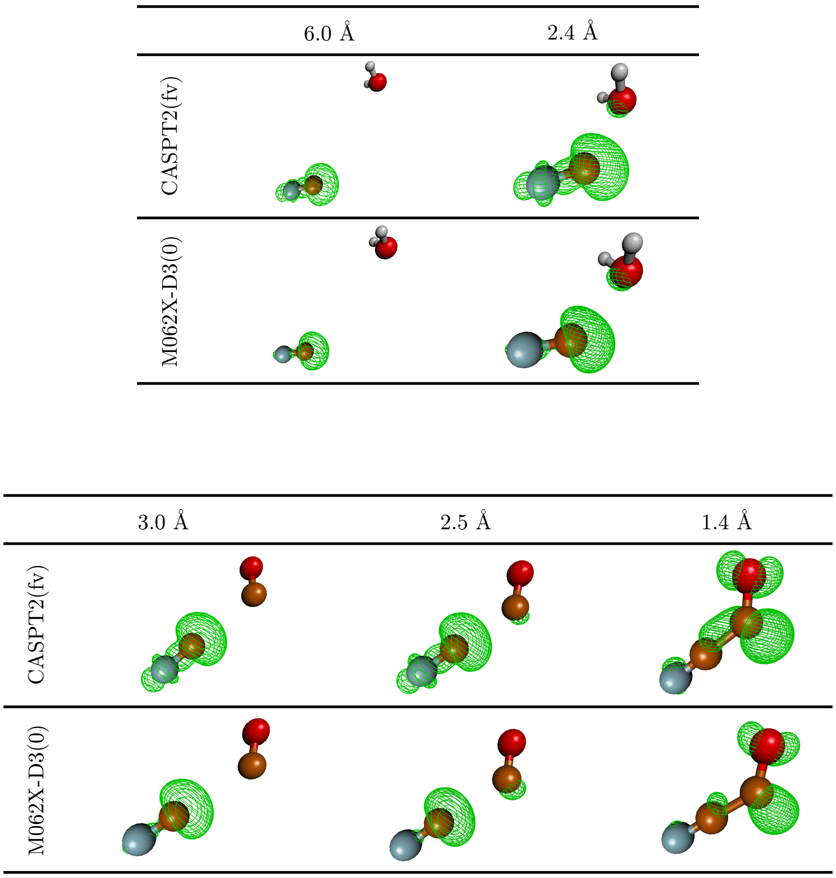

Figure 5: Evolution of the spin density along the formation of the CN−H_2O hemibond and along the formation of the NCCO radical at CASTP2(full-valence)/aug-cc-pVTZ and M062X-D3(0)/def2-TZVPD theory levels. Iso-surface level is 0.01, the same for all figures.

system along the range of C-C distances around the barrier location, from 2.0 to 4.0 Å, see Figure 6. The insets depict the spin density maps of the complex at each electronic state at the transition state distance of ∼2.5 Å.

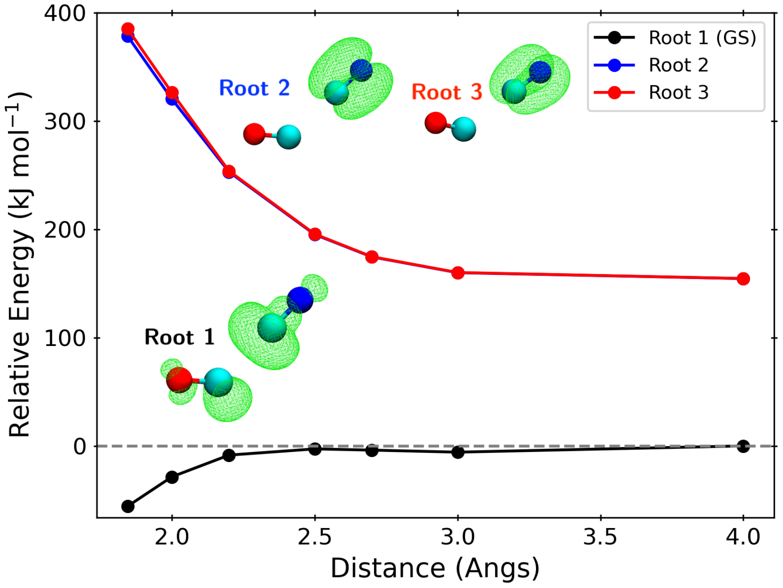

Figure 6: Potential energy surfaces for the three first states of NCCO around the barrier location. Energies were referred to the energy at 4.0 Åof the ground state (GS). The insets correspond to the spin densities of each root at 2.2 Å(i.e., just left from the barrier). They indicate where the unpaired electron is (isovalues are 0.05).

The first two excited states (roots 2 and 3) correspond to the swap of one of the electrons in the two π bonding orbitals of the CN group into the anti-bonding molecular orbital that hosts the unpaired electron in the ground state. All three states are doublets, with occupation values of 2210, 1220 and 2120 for roots 1-3, respectively, corresponding to the occupation of the 4 molecular orbitals: HOMO-2, HOMO-1, HOMO and LUMO (following the ordering of the ground state), see Figure 7. This confirms that there are no close-by electronic states interacting with the ground state potential energy surface at the region of the barrier, i.e., it is a real barrier.

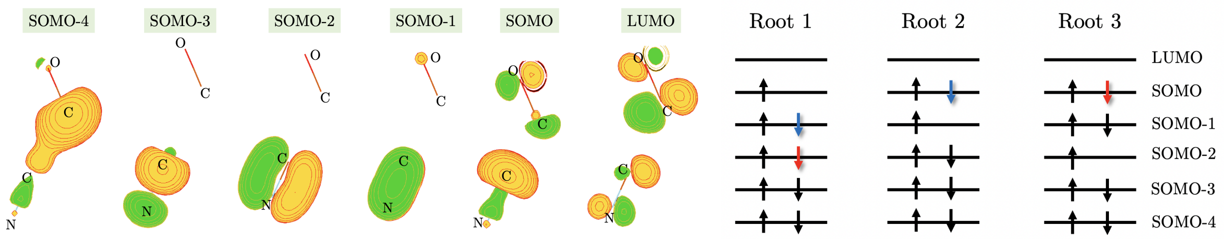

Figure 7: Left: Natural orbitals (standard output from OpenMolcas) of the NCCO system from the XMS-CASSCF calculation at 2.2 Å(isovalues=0.1). Right: ordering of electrons for the three first solutions. SOMO stands for "singly occupied molecular orbital" and LUMO for lowest unoccupied molecular orbital. Notice that the molecular orbital enumeration refers to the first root.

4 Reactivity on hemibonded CN-H2O

The benchmark data in Tab. 6 shows the good agreement between M062X-D3 and coupled cluster, which only deviate by 0.6 kJ mol^{-1}.

Table 6: Single point energy benchmark for $CN{-}H_2O_{hemi}$ + H_2 (on a single water molecule). R and TS are the electronic energies of reactants and the transition state in Hartree, respectively. $\Delta E^{\ddagger}$: electronic energy barriers in kJ mol^{-1}. Δ: energy difference between barriers at DFT and Coupled Cluster levels in kJ mol^{-1}. MUE(%): mean unsigned error. All DFT calculations are combined with a def2-TZVPD basis set, and CCSD(T)-F12 with AUG-CC-pVTZ. The initial geometries were generated at BHLYP-D4/def2-TZVPD level.

	R	TS	dE	Δ	MUE(%)
CCSD(T)-F12	-170.0892564	-170.0816774	19.9		
B3LYP-D4	-170.315019	-170.3086012	16.8	3.0	15.3
BHLYP-D4	-170.2924576	-170.2850424	19.5	0.4	2.2
M062X-D3	-170.3173162	-170.3095076	20.5	0.6	3.0
MPWB1K-D3(BJ)	-170.2949809	-170.2879311	18.5	1.4	7.0
PBE-D4	-170.2018696	-170.1978572	10.5	9.4	47.1
PW6B95-D4	-170.5914239	-170.584513	18.1	1.8	8.8
TPSSH-D3(BJ)	-170.4028249	-170.3963905	16.9	3.0	15.1
wB97m-D3(BJ)	-170.4314392	-170.4233951	21.1	1.2	6.1
wB97m-V	-170.323606	-170.3152324	22.0	2.1	10.5
wB97x-D4	-170.4365489	-170.4283031	21.6	1.8	8.8

5 Reactivity of NC···CO and NCCO

The benchmark data for the Van Der Waals complex NC···CO reactivity with H_2 is outlined in Table 8.

For the reaction of NCCO + H_2 $\rightarrow$ HCN + CO + H a very high energy barrier of 146.8 kJ mol^{-1} is found at M062X-D3/def2-TZVPD, and hence is not further considered and has been kept out of the study with large ice models. For the reaction of NCCO + H we found one transition state for the reaction H + NCCO towards HCN + CO, and one barrierless pathway towards HCOCN. This is summarized in Figure 8, with the raw data available in Table 7.

Finally, the NCCO + H_2 $\rightarrow$ HNCCO + H reaction, when tested at M062X-D3(0) sports a barrier of 231.4 kJ mol^{-1}, and hence was not included in the benchmark nor the general study. One other reaction was left out of this benchmark study: NCCO + H $\rightarrow$ HNCCO, because we could not converge the CCSD(T) calculations. However, given that M062X-D3(0)/def2-VTZPD has stood out in virtually all previous tests, will use this functional for this reaction on the larger ice models.

Table 7: Single point energy benchmark of reactions NCCO + H to form HCOCN and HCN + CO. DFT calculations where combined with the def2-VTZPD basis set, and coupled cluster ones with AUG-CC-PVTZ. Running an IRC from TS(HCN+CO) leads to HCOCN barrierlessly in the backwards direction. The initial geometries were generated at M062X-D3/def2-TZVPD level.

	Bare electronic energy (Hartree)				
NCCO + H	H asymp	CN-CO asymp	HCOCN	TS(HCN+CO)	HCN + CO
CCSD(T)-F12	-0.4998519374	-205.8320609	-206.4873181	-206.3253605	-206.4972567
B3LYP-D4	-0.4987642849	-206.0743022	-206.7226494	-206.5708624	-206.7256765
BHLYP-D4	-0.4985454281	-206.0560473	-206.7068695	-206.5497621	-206.7135662
M062X-D3	-0.4981407961	-206.0872363	-206.7396124	-206.5782874	-206.7477759
MPWB1K-D3(BJ)	-0.4978182571	-206.0686348	-206.7190453	-206.5602365	-206.7211898
PBE-D4	-0.4996179753	-205.9460273	-206.5897573	-206.4445247	-206.5818763
PW6B95-D4	-0.5013005495	-206.4190318	-207.0706796	-206.9154607	-207.073359
TPSSH-D3(BJ)	-0.4998536131	-206.1757608	-206.8258037	-206.6786623	-206.8246793
wB97m-D3(BJ)	-0.4986447077	-206.229025	-206.8818551	-206.720587	-206.8919437
wB97m-V	-0.4941160423	-206.1148633	-206.7631681	-206.6014974	-206.7721969
wB97x-D4	-0.5050291523	-206.2131331	-206.8708137	-206.7107885	-206.8761712

	Energies relative to the asymptote (kJ mol^{-1})		
	HCOCN	TS(HCN+CO)	HCN + CO
CCSD(T)-F12	-408.0	17.2	-434.1
B3LYP-D4	-392.7	5.8	-400.7
BHLYP-D4	-399.8	12.7	-417.4
M062X-D3	-404.9	18.6	-426.4
MPWB1K-D3(BJ)	-400.6	16.3	-406.3
PBE-D4	-378.4	2.9	-357.7
PW6B95-D4	-394.7	12.8	-401.8
TPSSH-D3(BJ)	-394.3	-8.0	-391.4
wB97m-D3(BJ)	-404.8	18.6	-431.3
wB97m-V	-404.8	19.6	-428.5
wB97x-D4	-400.8	19.4	-414.9

Table 8: Single point energy benchmark of NCCO + H_2 and NC$\cdots$CO (VdW) + H_2. VdW stands for Van der Waals interaction. DFT calculations were combined with the def2-VTZPD basis set, while the reference, CCSD(T)-F12, with AUG-CC-PVTZ. $\Delta E^{\ddagger}$ indicates barrier heights, while $\Delta E^{\ddagger}$ indicates reaction energies. MUE is the mean unsigned error, in percentage. Raw energies are gievn in Hartree and relative ones in kJ mol-1.

Raw energies	R (NCCO)	R (VdW)	TS	P
CCSD(T)-F12	-207.0079781	-206.9618183	-206.9559451	-206.9982887
B3LYP-D4	-207.2487264	-207.187548	-207.1886239	-207.2252854
BHLYP-D4	-207.2262247	-207.1674595	-207.1661433	-207.2116274
M062X-D3	-207.2569313	-207.2057099	-207.2010013	-207.2464236
MPWB1K-D3(BJ)	-207.2351648	-207.1727828	-207.1721203	-207.2191630
PBE-D4	-207.1146295	-207.0453124	-207.0492701	-207.0834903
TPSSH-D3(BJ)	-207.3569873	-207.2946616	-207.2954204	-207.3259167
wB97m-D3(BJ)	-207.4003759	-207.3452369	-207.3445405	-207.3912803
wB97m-V	-207.2769921	-207.2205967	-207.2198871	-207.2670637
wB97x-D4	-207.3962304	-207.3366812	-207.3361381	-207.3819985

ΔE	$\Delta E^{\ddagger}_{NCCO}$	ΔE^{RX}_{NCCO}	$\Delta E^{\ddagger}_{VdW}$	ΔE^{RX}_{VdW}
CCSD(T)-F12	136.6	25.4	15.4	-95.8
B3LYP-D4	157.8	61.5	-2.8	-99.1
BHLYP-D4	157.7	38.3	3.5	-116.0
M062X-D3	146.8	27.6	12.4	-106.9
MPWB1K-D3(BJ)	165.5	42.0	1.7	-121.8
PBE-D4	171.6	81.8	-10.4	-100.2
PW6B95-D4	160.9	52.1	0.0	-108.7
TPSSH-D3(BJ)	161.6	81.6	-2.0	-82.1
wB97m-D3(BJ)	146.6	23.9	1.8	-120.9
wB97m-V	149.9	26.1	1.9	-122.0
wB97x-D4	157.8	37.4	1.4	-119.0

MUE(%)	$\Delta E^{\ddagger}_{NCCO}$	ΔE^{RX}_{NCCO}	$\Delta E^{\ddagger}_{VdW}$	ΔE^{RX}_{VdW}
B3LYP-D4	15.5	141.9	118.3	3.5
BHLYP-D4	15.5	50.7	77.6	21.1
M062X-D3	7.5	8.4	19.8	11.6
MPWB1K-D3(BJ)	21.2	65.1	88.7	27.2
PBE-D4	25.6	221.4	167.4	4.7
PW6B95-D4	17.8	104.9	99.9	13.6
TPSSH-D3(BJ)	18.3	220.7	112.9	14.3
wB97m-D3(BJ)	7.3	6.1	88.1	26.2
wB97m-V	9.7	2.5	87.9	27.4
wB97x-D4	15.5	46.9	90.8	24.3

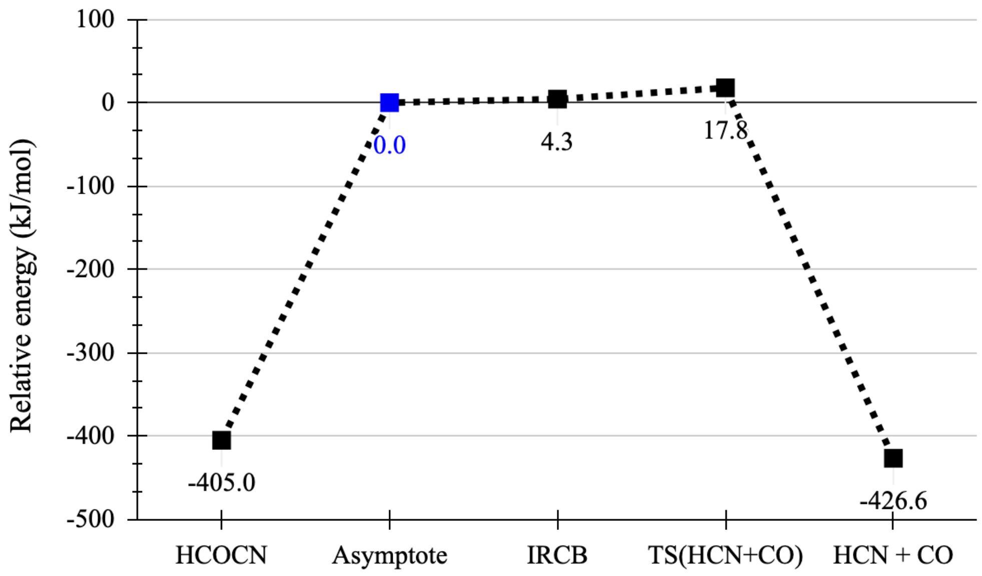

Figure 8: Energy scheme of the stationary points along the NCCO + H reaction including the asymptote (energy reference) and the last point of the backwards IRC calculation (IRCB) from the TS of NCCO + H → HCN + CO. Energies and geometries correspond to fully optimized structures (except for the IRCB point) at M062X-D3/def2-TZVPD level.

6 Gas-phase reaction $CN + H_2 \rightarrow HCN + H$

With an underestimation of 2.9 kJ mol^{-1} M062X-D3 is the best performing density functional.

Table 9: Single point energy benchmark for CN + H_2 → HCN + H reaction in the gas phase. R and TS are the electronic energies of reactants and the transition state in Hartree, respectively. $\Delta E^{\ddagger}$: electronic energy barriers in kJ mol^{-1}. Δ: energy difference between barriers at DFT and Coupled Cluster levels in kJ mol^{-1}. MUE(%): mean unsigned error. All DFT calculations are combined with a def2-TZVP basis set, and CCSD(T)-F12 with AUG-CC-pVTZ. The initial geometries were generated at BHLYP-D3(BJ)/def2-TZVP level.

	R	TS	$\Delta E^{\ddagger}$	Δ	MAE(%)
CCSD(T)-F12	-93.77340211	-93.76844977	13.0		
B3LYP-D4	-93.87473703	-93.87498656	-0.7	13.7	105.0
BHLYP-D4	-93.86069359	-93.85899763	4.5	8.5	65.8
M062X-D3	-93.88119745	-93.87734363	10.1	2.9	22.2
MPWB1K-D3(BJ)	-93.86107908	-93.86008514	2.6	10.4	79.9
PBE-D4	-93.80830099	-93.81087417	-6.8	19.8	152.0
PW6B95-D4	-94.03727998	-94.03690313	1.0	12.0	92.4
TPSSH-D3(BJ)	-93.93264028	-93.93262149	0.0	13.0	99.6
wB97m-D3(BJ)	-93.94478385	-93.94353153	3.3	9.7	74.7
wB97m-V	-93.88000838	-93.87877301	3.2	9.8	75.1
wB97x-D4	-93.94899724	-93.94781687	3.1	9.9	76.2

7 NCCO formation on the surface

The geometries of the PES' stationary points on the 13 CO ice model are shown in Figure 9. The ZPE-corrected energy barrier is 2.5 kJ mol^{-1}, and the reaction energy is -125.7 kJ mol^{-1}. The transition state has an imaginary frequency of 122.8 cm^{-1}. Taking the asymptote CN + $(CO)_{13}$ as the energy reference, the ZPE-corrected reaction energy is 134.9 kJ mol^{-1}. A table with the energetics is available below.

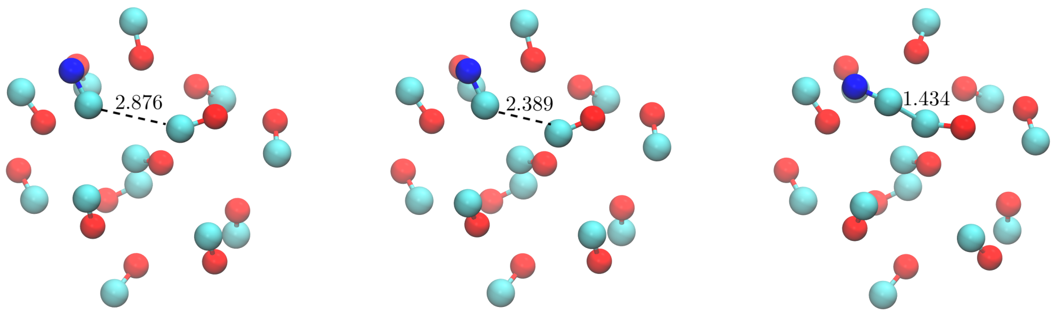

Figure 9: Reaction steps (reactants, transition state and product) to form NCCO from a Van der Waals-complexed CN to CO. Distances in Å.

Table 10: Energetics of NCCO formation on $(CO)_{13}$ ice. Bare energies in Hartree, relative energies in kJ mol^{-1}.

	E	ZPE	ΔE	Δ(E+ZPE)
React	-1565.930321	0.08189626		
TS (122.84)	-1565.929451	0.08198308	2.3	2.5
Prod	-1565.981463	0.08516130	-134.3	-125.7

8 Kinetics calculations

We have employed microcanonical transition state theory and the Eckart barrier to include quantum tunneling effects model to obtain the rate constants of some of the reactions studied in this work with the QuantumGrain kinetics code.[6] All reaction rate constants benefit from tunneling, deviating their rate constants from linearity in the Arrhenius plot (see the main text) as expected according to the crossover temperatures.These rate constants are unimolecular, i.e., the system comprised by the cluster and reactants is treated as a "large molecule" undergoing an intramolecular chemical reaction. Notice that while the Eckart formulation is a very good one-dimensional model able to reach great level of accuracy (see e.g.[7]), it may still be not as good as much more expensive multidimensional alternatives, such as Instanton theory.[8]

The Kinetic Isotope Effects (KIE) of these reactions were also calculated and are shown in Figure 10. The KIE is the ratio of the rate constant for the reaction with the light isotopologue divided by the value for the reaction with, heavier one, in this case deuterium

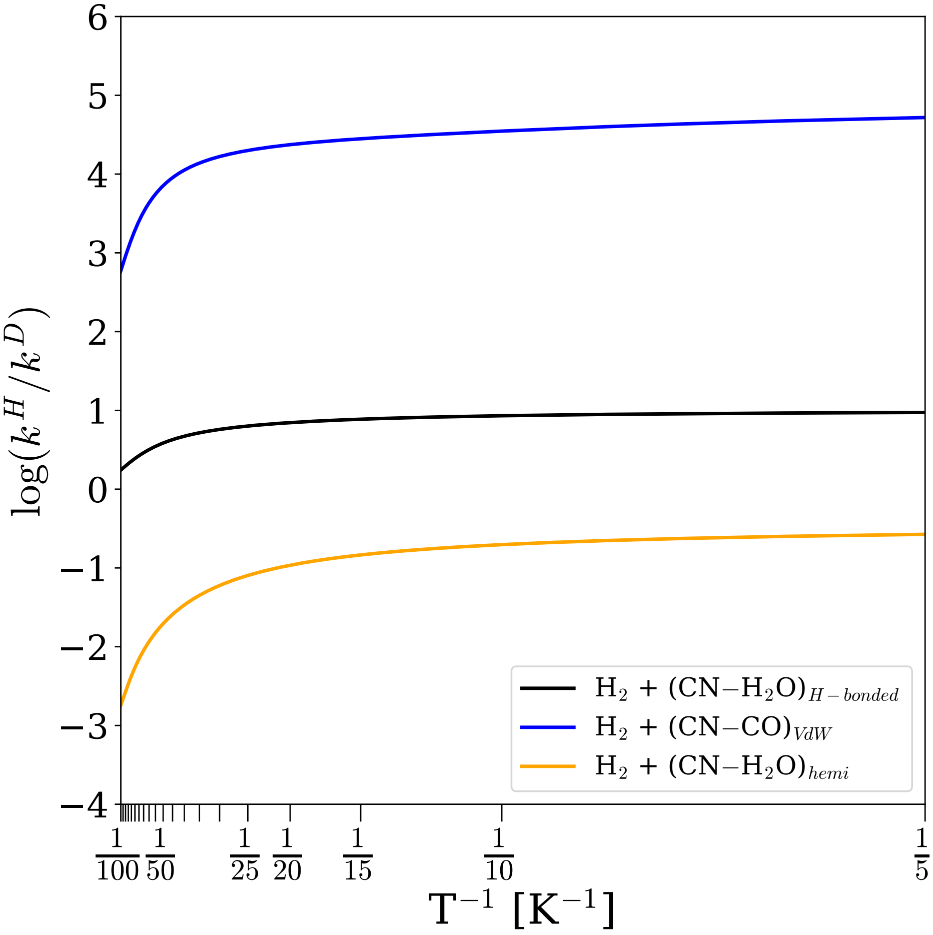

Figure 10: Kinetic Isotopic Effects of the three $H_2(HD) + CN$ reactions studied in the main text.

9 Binding energy tables

Table 11: Raw energies of bare ices and CN used in the calculation of binding energy distributions. Energies in Hartree.

Bare ices and CN		
	DFT	ZPE
CN	-92.711933	0.005121
$(CO)_{13}$	-1473.212694	0.074568
$(CO)_{18}$	-2039.834729	0.103799
$(H_2O)_{14}$	-1070.229472	0.356068
$(H_2O)_{18}$	-1376.017320	0.459329

Table 12: Raw energies of CN complexes with water and CO ices used in the calculation of binding energy distributions. Energies in Hartree.

$CN\cdots(CO)_{13}$		$CN\cdots(CO)_{18}$		$CN\cdots(H_2O)_{14}$		$CN\cdots(H_2O)_{18}$	
DFT	ZPE	DFT	ZPE	DFT	ZPE	DFT	ZPE
-1565.929973	0.080348	-2132.551702	0.108921	-1162.957455	0.361497	-1468.759432	0.466195
-1565.930640	0.081875	-2132.548308	0.108203	-1162.957744	0.361745	-1468.746409	0.465796
-1565.930560	0.081484	-2132.550914	0.108958	-1162.958137	0.362738	-1468.749133	0.467412
-1565.929584	0.081996	-2132.550936	0.108531	-1162.957630	0.362762	-1468.746089	0.465053
-1565.929728	0.081579	-2132.550936	0.108727	-1162.959800	0.362780	-1468.749914	0.464937
-1565.930582	0.081881	-2132.550936	0.108117	-1162.957760	0.361300	-1468.748817	0.465281
-1565.927824	0.080877	-2132.550936	0.108849	-1162.957573	0.362250	-1468.744249	0.466915
-1565.930085	0.081467	-2132.550936	0.110729	-1162.957548	0.361665	-1468.740944	0.465847
-1565.930458	0.080843	-2132.551245	0.106847	-1162.957566	0.362578	-1468.756231	0.465531
-1565.930231	0.081259	-2132.551982	0.108674	-1162.961880	0.361480	-1468.746065	0.465342
-1565.929396	0.081439	-2132.550886	0.107298	-1162.946301	0.362509	-1468.746577	0.464836
-1565.930124	0.081739	-2132.551088	0.109138	-1162.959785	0.362791	-1468.750069	0.465904
		-2132.551734	0.108912	-1162.947615	0.362643	-1468.753677	0.466124
		-2132.549640	0.108758	-1162.963946	0.361833	-1468.751104	0.463928
		-2132.550830	0.108026	-1162.959857	0.363153	-1468.755500	0.465359
		-2132.551720	0.109597	-1162.960168	0.362506	-1468.740702	0.463851
		-2132.552712	0.108826	-1162.965350	0.362217	-1468.734192	0.465401
		-2132.551871	0.108072	-1162.960261	0.362761	-1468.755130	0.465943
		-2132.547963	0.108370	-1162.964591	0.363356	-1468.753616	0.466290
		-2132.551792	0.109457	-1162.963936	0.361773	-1468.734629	0.465620
		-2132.550385	0.108228	-1162.947275	0.362430	-1468.749488	0.465984
		-2132.552044	0.107950	-1162.960891	0.363192	-1468.734246	0.465881
				-1162.960256	0.362664	-1468.753065	0.465701
				-1162.964498	0.363143	-1468.733122	0.465545
				-1162.958797	0.362709	-1468.753636	0.466258
				-1162.958176	0.362482	-1468.749473	0.466154
						-1468.734624	0.465440

References

(1) Wakelam, V.; Loison, J. C.; Mereau, R.; Ruaud, M. Binding energies: New values and impact on the efficiency of chemical desorption. *Mol. Astrophys.* **2017**, *6*, 22–35.

(2) Garrod, R. T.; Herbst, E. Formation of methyl formate and other organic species in the warm-up phase of hot molecular cores. *Astron. Astrophys.* **2006**, *457*, 927–936.

(3) Belloche, A.; Garrod, R. T.; Müller, H. S. P.; Menten, K. M. Detection of a branched alkyl molecule in the interstellar medium: iso-propyl cyanide. *Science* **2014**, *345*, 1584–1587.

(4) Lamberts, T.; Markmeyer, M. N.; Kolb, F. J.; Kästner, J. Formation of Acetaldehyde on CO-Rich Ices. *ACS Earth Space Chem.* **2019**, *3*, 958–963.

(5) Yu, G.-t.; Ding, Y.-h.; Huang, X.-r.; Bai, H.-t.; Sun, C.-c. Theoretical Study on Stability and Properties of NC_2O Isomers. *J. Phys. Chem. A* **2005**, *109*, 2364–2372.

(6) Enrique-Romero, J.; Rimola, A. QuantumGrain RRKM code. `https://zenodo.org/doi/10.5281/zenodo.10518615`.

(7) Senevirathne, B.; Andersson, S.; Dulieu, F.; Nyman, G. Hydrogen atom mobility, kinetic isotope effects and tunneling on interstellar ices (I_h and ASW). *Mol. Astrophys.* **2017**, *6*, 59–69.

(8) McConnell, S.; Kästner, J. Instanton rate constant calculations close to and above the crossover temperature. *J. Comput. Chem.* **2017**, *38*, 2570–2580.
